# A functional genomics approach to dissect spotted alfalfa aphid resistance in *Medicago truncatula*

**DOI:** 10.1038/s41598-020-78904-z

**Published:** 2020-12-17

**Authors:** Silke Jacques, Jana Sperschneider, Gagan Garg, Louise F. Thatcher, Ling-Ling Gao, Lars G. Kamphuis, Karam B. Singh

**Affiliations:** 1 CSIRO Agriculture and Food, Floreat, WA 6014 Australia; 2 Centre for Crop and Disease Management, Curtin University, Bentley, WA 6102 Australia; 3 Biological Data Science Institute, The Australian National University, Canberra, ACT 2600 Australia; 4 CSIRO Agriculture and Food, Canberra, ACT 2601 Australia; 5 The UWA Institute of Agriculture, University of Western Australia, Crawley, WA 6009 Australia

**Keywords:** Plant sciences, Plant stress responses, Herbivory

## Abstract

Aphids are virus-spreading insect pests affecting crops worldwide and their fast population build-up and insecticide resistance make them problematic to control. Here, we aim to understand the molecular basis of spotted alfalfa aphid (SAA) or *Therioaphis trifolii* f*. maculata* resistance in *Medicago truncatula*, a model organism for legume species. We compared susceptible and resistant near isogenic *Medicago* lines upon SAA feeding via transcriptome sequencing. Expression of genes involved in defense and stress responses, protein kinase activity and DNA binding were enriched in the resistant line. Potentially underlying some of these changes in gene expression was the finding that members of the MYB, NAC, AP2 domain and ERF transcription factor gene families were differentially expressed in the resistant versus susceptible lines. A TILLING population created in the resistant cultivar was screened using exome capture sequencing and served as a reverse genetics tool to functionally characterise genes involved in the aphid resistance response. This screening revealed three transcription factors (a NAC, AP2 domain and ERF) as important regulators in the defence response, as a premature stop-codon in the resistant background led to a delay in aphid mortality and enhanced plant susceptibility. This combined functional genomics approach will facilitate the future development of pest resistant crops by uncovering candidate target genes that can convey enhanced aphid resistance.

## Introduction

Aphids are the most economically important sap-sucking insect pests worldwide, causing yield losses due to direct feeding damage and as transmitters of over 50% of all plant viruses^1,2^. Aphids reproduce asexually via parthenogenesis making them clonal in nature which allows them to build up population numbers fast and efficiently^3^. Current aphid control management mainly relies on the frequent use of insecticides in the field. This, in combination with the fast reproduction rate of aphids, has resulted in aphid resistance to multiple insecticide classes, thereby increasing the cost and difficulty to control them^4^. The emergence of insecticide resistance and imposed restrictions on usage of pesticides has greatly increased the need for novel and sustainable aphid control strategies. However, to develop such strategies there is a pressing need to gain a better understanding of the molecular basis of plant–aphid interactions and thus a need for fundamental research on the molecular mechanisms involved in effective defence against these destructive pests.

*Therioaphis trifolii* f*. maculata* or spotted alfalfa aphid (SAA) is a threat to legumes worldwide and is a renowned pest of lucerne (alfalfa; *Medicago sativa*). SAA carries and transmits amongst others the alfalfa mosaic virus (AMV) and bean yellow mosaic virus (BYMV), two common viruses causing destructive losses to lucerne and other crops^5^. Lucerne or alfalfa is a perennial legume fodder and forms the backbone of large livestock industries worldwide as a major pasture crop^6^. Spain and the United States of America are the top two exporters of alfalfa meal and pellets with a ten year average of 312 and 229 kilotonnes of alfalfa (FAOSTAT). *Medicago truncatula* Gaertn (barrel medic) is a cultivated pasture species and a close relative of *M. sativa* as demonstrated by their genome-scale synteny with marker relationships uniformly syntenic^7^. With *M. truncatula* being a model organism for legumes, it has excellent resources for functional genomics studies, including a genome sequence and a database with integrated tools for genome browsing and data mining^8^. *M. truncatula* is a host for SAA and other aphids, and germplasm accessions with natural genetic resistance to a range of aphid species have been identified^9^, making it an excellent system to study plant-aphid interactions. The large scale synteny among legumes signifies that knowledge gained about plant defense mechanisms against aphids from the *M. truncatula* model system could be translated to other legume species^10^.

Recurrent backcrossing of an aphid-resistant donor line SA2927^11^ to the reference genotype of *M. truncatula,* Jemalong (A17) created the cultivar Jester, which is resistant to SAA but also to bluegreen aphid (*Acyrthosiphon kondoi*) and pea aphid (*Acyrthosiphon pisum*)^12^. The susceptible A17 and resistant Jester are near isogenic lines and share about 90% sequence similarity making them valuable for the study of plant-aphid interactions^13^. The *M. truncatula*-aphid system has been vital for the considerable progress made over the last few years on the plant side of the interaction with the identification of single dominant resistance genes and/or quantitative trait loci (QTLs) against bluegreen, cowpea and pea aphid as well as SAA and progress on downstream defence mechanisms against bluegreen and pea aphid^14–16^.

*R*-gene mediated resistance against SAA in Jester is caused by the single dominant locus *TTR* (*Therioaphis trifolii* resistance) on the long arm of chromosome three^17^. Although *TTR* is linked to the BGA resistance gene on chromosome 3 (*AKR*), it acts independently to reduce the survival rate of SAA. Feeding of SAA on a susceptible *M. truncatula* line lacking the *TTR* gene, leads to a striking phenotype of systemically induced vein chlorosis^17^. *TTR* is a particularly strong acting aphid resistance gene with 80% of aphids feeding on the resistant Jester line dead within 24 h and all aphids dead within 48 hrs^17^. However, the underlying molecular mechanics and downstream signalling events of SAA resistance remain elusive yet would enhance our understanding of the molecular basis of plant interaction with phloem-feeding insects. New insights into plant mediated resistance to SAA will provide information for designing and engineering new and more effective resistance and/or improved strategies to protect crops from these destructive aphid insect pests.

Next-generation sequencing has progressed transcriptomics at an unprecedented speed, at relatively low cost and thereby facilitating routine quantitative transcriptome profiling^18^. RNA sequencing (RNA-seq) is a short read massively parallel sequencing methodology based on a variation of pyrosequencing and allows for the quantification of RNA in a biological sample at a given point in time. By choosing multiple time points, the genome-wide transcriptomic changes can be captured and the dynamic landscape of gene regulation untangled.

Another useful tool in functional genomics is the creation of TILLING (targeting induced local lesions in genomes) populations. The TILLING approach is based on the generation of genome-wide single nucleotide changes, using a chemical mutagen such as ethyl methanesulfonate (EMS), followed by a screening method to identify individual mutant lines carrying a mutation in genes of interest^19^. The individual SNP changes caused by mutagenesis can affect a range of protein functions, e.g. alter important catalytic or interaction residues, altered splicing or generate a truncated protein. The main advantage of TILLING as a reverse genetics strategy is that it can be applied to any species, irrespective of genome size and ploidy and is considered a non-genetically modified technique. Identification of SNP changes in the gene sequences of a TILLING population can be achieved in various ways. A popular approach to catalogue mutations is the use of exome capture, a reduced representation approach to capture the gene-coding sequences for a species’ genome^20^. This can be developed for the full gene coding sequences or a subset of genes that one would be interested in^21^, thus leading to significant cost reductions and computational data processing costs, whilst identifying individuals with mutations in the genes of interest for a given study^22^.

Despite the economic importance of SAA, very little is known of the downstream molecular mechanisms underlying its resistance in legumes. In this study, we made use of resistant (Jester) and susceptible (A17) near isogenic *M. truncatula* lines to SAA as a model to study plant-SAA interactions. We sequenced plant transcriptomes in response to SAA feeding and identified genes that are differentially expressed between resistant and susceptible *M. truncatula* cultivars. Quantitative reverse transcription polymerase chain reaction (qRT-PCR) of six randomly selected transcription factor encoding genes representing major transcription factor classes validated the reliability of our RNA-seq results. Furthermore, we created a TILLING population in the resistant *M. truncatula* cultivar Jester as a reverse genetics tool to functionally characterise genes involved in the resistance response. The TILLING population was screened for candidates carrying a premature stop-codon in a subset of transcription factor genes identified as differentially regulated by SAA. Three transcription factors were shown to be important regulatory genes in the defence response to SAA as a premature stop-codon in a Jester background led to a delay in SAA mortality and enhanced plant susceptibility. We believe unravelling the transcriptomic footprint of plant resistance in combination with screening a TILLING population will enable a better understanding of the molecular basis of aphid resistance. This can uncover candidate gene targets for future use in the development of pest resistant crops thereby minimizing the dependency of chemical pesticides.

## Results

### Transcriptomic profiling of resistant and susceptible *M. truncatula* cultivars upon SAA infestation

To identify genes that are differentially expressed during aphid predation, we made use of two near isogenic lines of *M. truncatula*; Jester and A17. Jester is resistant to SAA and carries the *TTR* resistance gene, whilst A17 does not and is susceptible to SAA. To investigate the differences of gene regulation in response to SAA feeding, two single trifoliate leaves from Jester and A17 plants were either non-infested or each infested with 20 late instar/adult SAA and leaves were harvested at two time points, namely after 12 and 24 h (h) of SAA feeding. As SAA adults die within 24–48 h on Jester plants we selected the 12 and 24 h time points to compare and contrast the molecular responses of the two *M. truncatula* near isogenic lines. The transcriptional response to aphid infestation at each time point was compared to the non-infested controls and comparisons were also made between resistant and susceptible lines. Prior to RNA-sequencing, validation of aphid infestation was performed by analysing defence marker genes for the salicylic acid (SA) and jasmonic acid (JA) pathways, using qPCR^23^ (Supplementary Fig. 1). Subsequent RNA-seq of infested or non-infested leaves of Jester and A17 plants after 12 and 24 h of SAA feeding showed that for each sample over 90% of RNA sequence reads aligned to the *M. truncatula* genome (Table 1).

**Table 1:** RNA sequencing read numbers and mapping rate to *M. truncatula.*

Sample name*	Total reads	Mapping rate (%)
ANI-12-1	31,773,251	90.77
ANI-12-2	39,771,495	91.20
ANI-12-3	34,988,340	91.37
ANI-24-1	42,186,258	91.79
ANI-24-2	47,425,859	91.83
ANI-24-3	52,322,870	91.66
ASA-12-1	44,818,009	91.54
ASA-12-2	38,209,784	91.51
ASA-12-3	42,082,284	91.02
ASA-24-1	52,272,350	91.72
ASA-24-2	48,724,576	91.70
ASA-24-3	48,171,066	91.30
JNI-12-1	37,492,453	91.04
JNI-12-2	41,969,246	91.39
JNI-12-3	39,238,961	91.31
JNI-24-1	40,157,915	92.38
JNI-24-2	43,635,907	92.10
JNI-24-3	51,552,489	92.28
JSA-12-1	40,410,236	91.34
JSA-12-2	47,415,106	91.47
JSA-12-3	36,472,271	91.73
JSA-24-1	43,049,171	91.37
JSA-24-2	48,327,849	91.50
JSA-24-3	44,447,121	91.24

*A, A17; J, Jester; NI, non infested; SA, SAA infested; 12, after 12 h infestation; 24, after 24 h infestation; -1/-2/-3, number of biological repeat.

**Figure 1:**
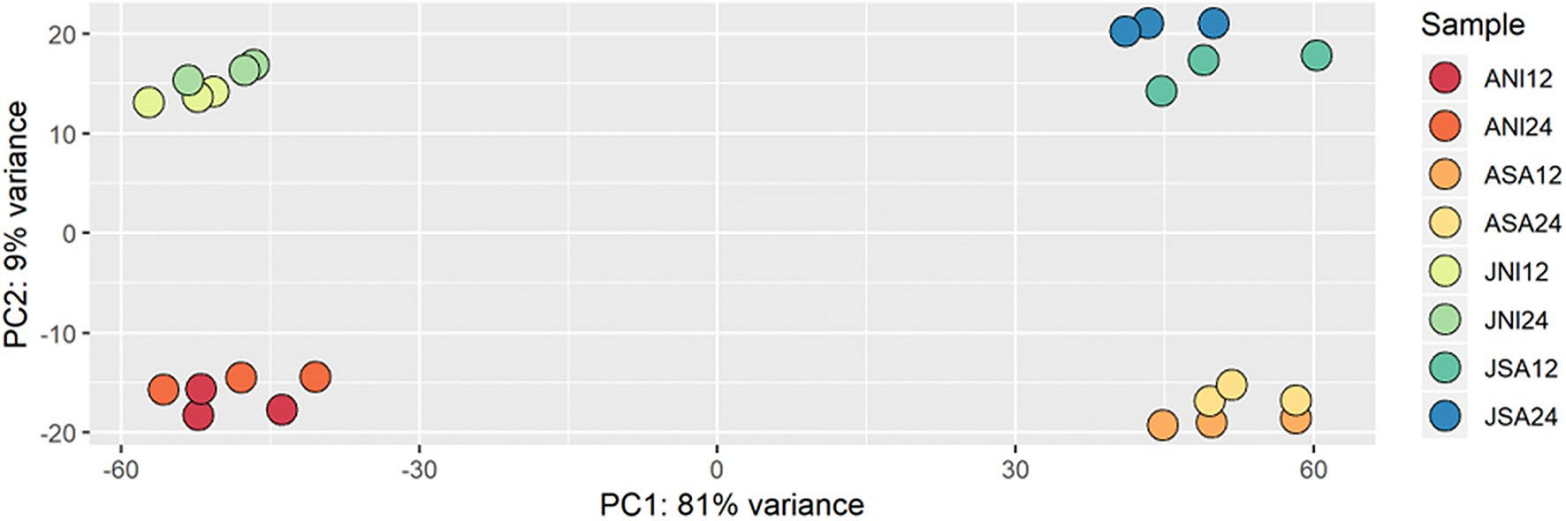
PCA plot shows distinct clusters of SAA infested and non-infested *M. truncatula* Jester and A17 samples. Principal component analysis of the 24 RNA sequencing samples exposes clustering of the three biological repeats per treatment and genotype. The infested (-SA) and non-infested (-NI) samples between two time-points (12 or 24) are grouped together. There was less variance between Jester infested (JSA12 and JSA 24) and A17 infested samples (ASA12 and ASA24) compared to their non-infested counterparts JNI12, JNI24 and ANI12, ANI24 respectively.

Principal component analysis shows the RNA-seq samples cluster together per treatment and genotype (Fig. 1). At either time point, when compared to their non-infested controls both Jester and A17 had over 14,000 genes differentially regulated upon SAA infestation, which corresponds to more than 22% of all annotated *Medicago* genes (Table 2). The number of activated and repressed genes in response to SAA feeding at a given time point in a given cultivar were comparable and ranged from 6890 down-regulated genes after 24 h in Jester to 8066 up-regulated genes in Jester after 12 h. Thus, both in the resistant Jester and susceptible A17 plants there is no major variation in the number of differentially expressed genes (DEGs) compared to non-infested controls over time (Table 2). This is in contrast to comparisons between Jester and A17 infested samples with a strong increase in the number of DEGs over time. A total of 5407 genes were differentially expressed between Jester and A17 after 24 h of SAA feeding compared to only 2480 DEGs after 12 h of SAA feeding (Table 2).

**Table 2:** Differentially expressed genes (DEGs) between resistant Jester and susceptible A17 *M. truncatula* varieties upon 12 and 24 h of SAA infestation.

DEGs in treatment comparisons	Up-regulated	Down-regulated	Total
Jester SAA 12 h—Jester non-infested 12 h	8066	7369	15,435
Jester SAA 24 h—Jester non-infested 24 h	7312	6890	14,202
A17 SAA 12 h—A17 non-infested 12 h	7516	7104	14,620
A17 SAA 24 h—A17 non-infested 24 h	7573	7208	14,781
Jester SAA 12 h—A17 SAA 12 h	1317	1163	2480
Jester SAA 24 h—A17 SAA 24 h	2490	2917	5407

**Figure 2:**
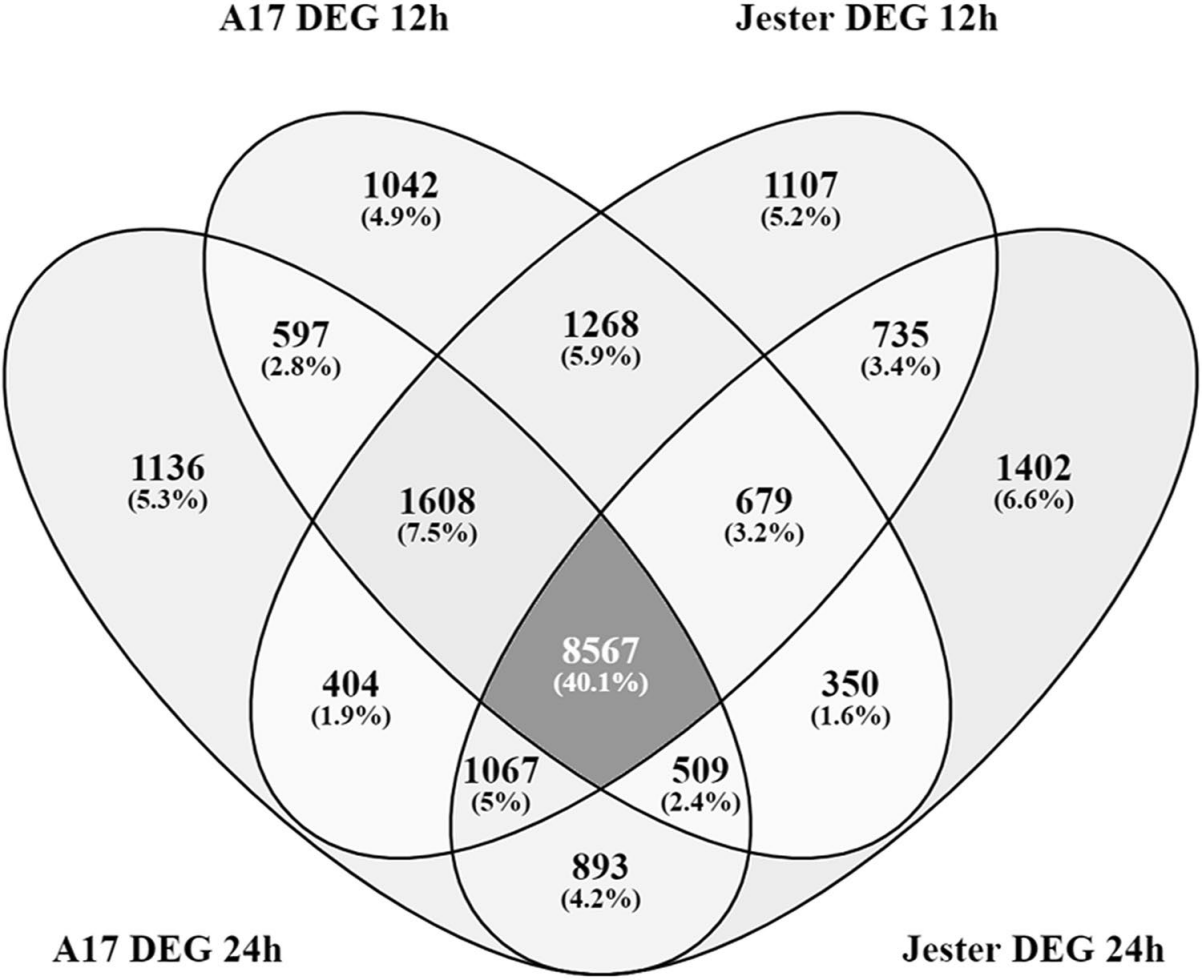
Venn diagram of differentially expressed genes in *M. truncatula* cultivars Jester and A17, 12 and 24 h post treatment (un-infested or SAA infested). Differentially expressed genes (DEGs) between the infested and non-infested control for a given cultivar and time point are compared. A core set of 8,567 genes (40%) are regulated in response to SAA feeding irrespective of the genotype (Jester and A17).

To investigate the nature and the dynamics of the transcriptomic changes upon aphid predation, we compared the DEGs of resistant Jester and susceptible A17 lines at both time points (Fig. 2, Supplementary Tables 1 and 2). There is a core set of 8,567 DEGs overlapping between Jester and A17 compared to their non-infested controls. This corresponds to 40% of all differentially expressed genes shared in response to SAA feeding, regardless of genotype or time (Fig. 2). Even at a given time point, there is more than 50% of DEGs in common between aphid infested resistant and susceptible plants.

### Gene ontology enrichment and pathway involvement

**Figure 3:**
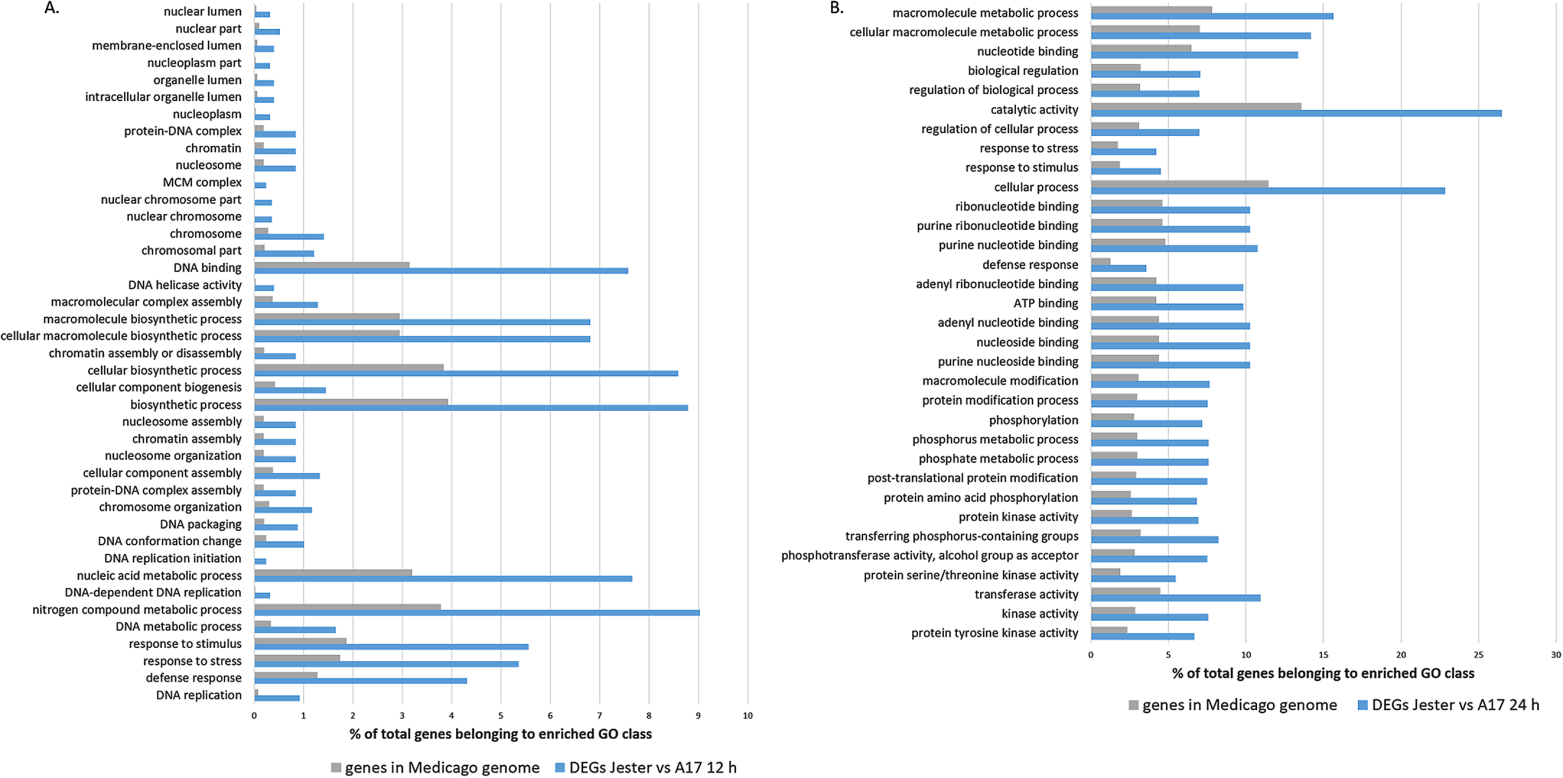
Gene ontology enrichment of differentially regulated genes of *M. truncatula* cultivars Jester and A17 following infestation with SAA. Gene ontology enrichment study was performed using agriGO on the DEGs between Jester and A17 at 12 h (**A**) and 24 h (**B**) of SAA infestation. The graphical representation compares the percentage of genes belonging to a significant enriched GO term (*P* < 0.001) in our DEGs dataset to the percentage of genes of the *M. truncatula* genome fitting this GO class.

To explore the differences between a resistant and susceptible defence response to SAA, we performed a gene ontology (GO) enrichment study using agriGO^24^ of the DEGs between Jester and A17 at 12 h and 24 h for the three existing GO categories, namely biological process, molecular function and cellular component. At 12 h there is a significant GO term enrichment of genes involved in defense response, response to stress and stimulus as well as genes involved in DNA replication and nucleosome assembly for the GO category biological process (Fig. 3A). For cellular component, there is an enrichment in genes located in the nuclear chromosome part, the nucleoplasm and mini chromosome maintenance (MCM) complex whilst for the molecular function, genes with DNA binding and DNA helicase activity were enriched in the differentially expressed gene set between Jester and A17 at 12 h (Fig. 3A). After 24 h of SAA feeding, the GO enriched terms shift to ATP binding and protein kinase activity (serine/threonine and tyrosine) for molecular function and cytoplasm is an enriched cellular component (Fig. 3B). Whilst there is still an enrichment of genes involved in defence response, phosphorylation and post-translational protein modification are also now enriched in biological processes (Fig. 3B).

**Figure 4:**
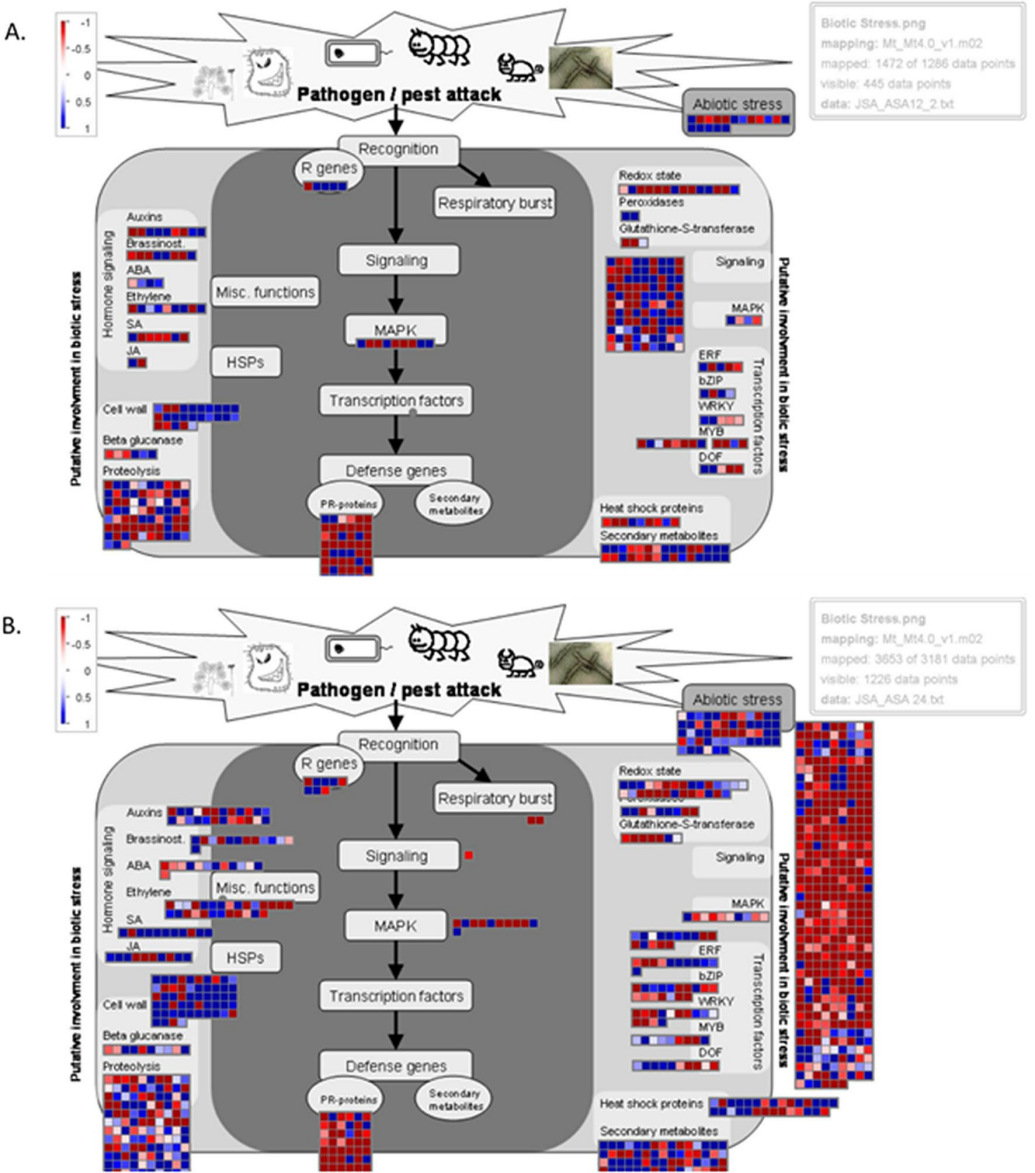
Graphical representation of regulated *M. truncatula* genes involved in the SAA defence response. DEGs between Jester and A17 at both 12 h (**A**) and 24 h (**B**) are plotted on a biotic stress infographic using Mapman analysis^25^. Activated genes are shown as blue squares whilst repressed genes in Jester compared to A17 are shown in red as indicated by the colour scale on the top left (LOG_2_ fold change). The defense response intensifies over time with the number of signaling genes increasing, albeit with the majority of them down-regulated in Jester (**B**).

Next, we made use of MapMan software^25^ to display the DEGs between Jester and A17 onto diagrams of metabolic pathways. Figure 4 illustrates the order of events when a plant cell is under attack where each square represents a DEG and is colour-coded according to up- or down regulation. The differences between Jester and A17 response to SAA feeding intensifies over time as becomes apparent when comparing the plots of DEGs between Jester and A17 at 12 h (Fig. 4A) and 24 h (Fig. 4B). This comparison demonstrates that the number of DEGs between resistant Jester and susceptible A17 cultivars almost doubles after 24 h compared to 12 h of SAA feeding. Whilst 2,490 genes are activated in Jester compared to A17, 2,917 genes are repressed, bringing the total of differentially regulated genes between Jester and A17 to 5,407 after 24 h of SAA feeding whereas at 12 h the total number of DEGs is 2,480 (Supplementary Tables 1 and 2). Hormonal regulation changes observed involves genes encoding members of the jasmonic acid, salicylic acid, ethylene and to a lesser degree abscisic acid signalling pathways and one or more of these pathways have been associated with plant defense to specific aphids^23,26,27^. Interestingly, this study also found that genes regulating auxin signalling are differentially expressed in a resistant but not the susceptible genotype during SAA predation. At 12 h, there are 27 genes regulated that are either auxin-induced or involved in auxin transport, whilst this number increases to 51 genes after 24 h. Cell wall related genes also play a role in fending off the pest attack as becomes apparent in the various up-regulated genes in resistant Jester compared to susceptible A17. Signalling genes and MAPK and transcription factor encoding genes also appear to be crucial for a resistant response with numbers increasing over time in Jester when compared to the susceptible A17 (Fig. 4B).

### Transcription factor profiling

**Figure 5:**
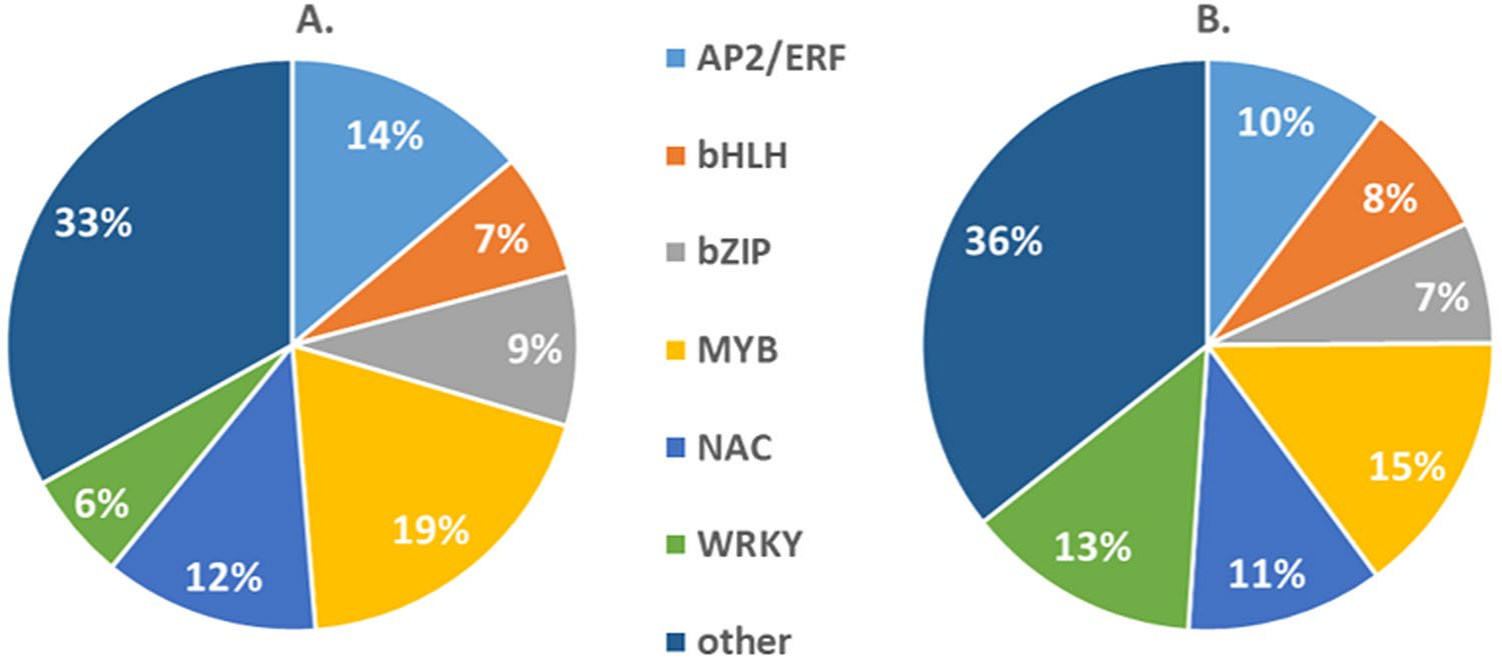
MYB TFs most regulated family over time upon SAA feeding. The most abundant transcription factor classes are shown with their percentages of DEGs after 12 h (**A**) and 24 h (**B**) of SAA feeding. The WRKY TF class undergoes the biggest shift over time, with a more than two-fold increase at 24 h.

Transcription factors play a vital role in regulating the transcriptomic changes during plant stress responses and are important signal transducers^28^. We therefore investigated different classes of transcription factors (TFs) that are regulated differently (*P* < 0.01) between Jester and A17 following SAA infestation. A total of 115 TFs are regulated after 12 h of SAA feeding which increases to 233 TF at 24 h. Percentage wise, this correlates to 4.5% of the total DEGs between Jester and A17 for both time points. After 12 h of SAA feeding, 19% of differentially regulated TFs belong to the MYB family making it the major class of differentially regulated TFs (Fig. 5A). Second with 14% are TFs of the APETALA 2/ETHYLENE RESPONSIVE FACTOR (AP2/ERF) family followed by the NAC TFs which make up 12% (Fig. 5A). Although the MYB family still makes up the largest portion of DEG TFs at 24 h, there is a 4% decrease, which is also the case for the AP2/ERF family members who drop to fourth largest class of differential regulators between Jester and A17 (Fig. 5B). Interestingly, the WRKY family of TFs shifts over time with an increase of 7%, making it the second largest class of TFs differentially regulated between resistant and susceptible plants after 24 h of SAA infestation (Fig. 5B).

**Figure 6:**
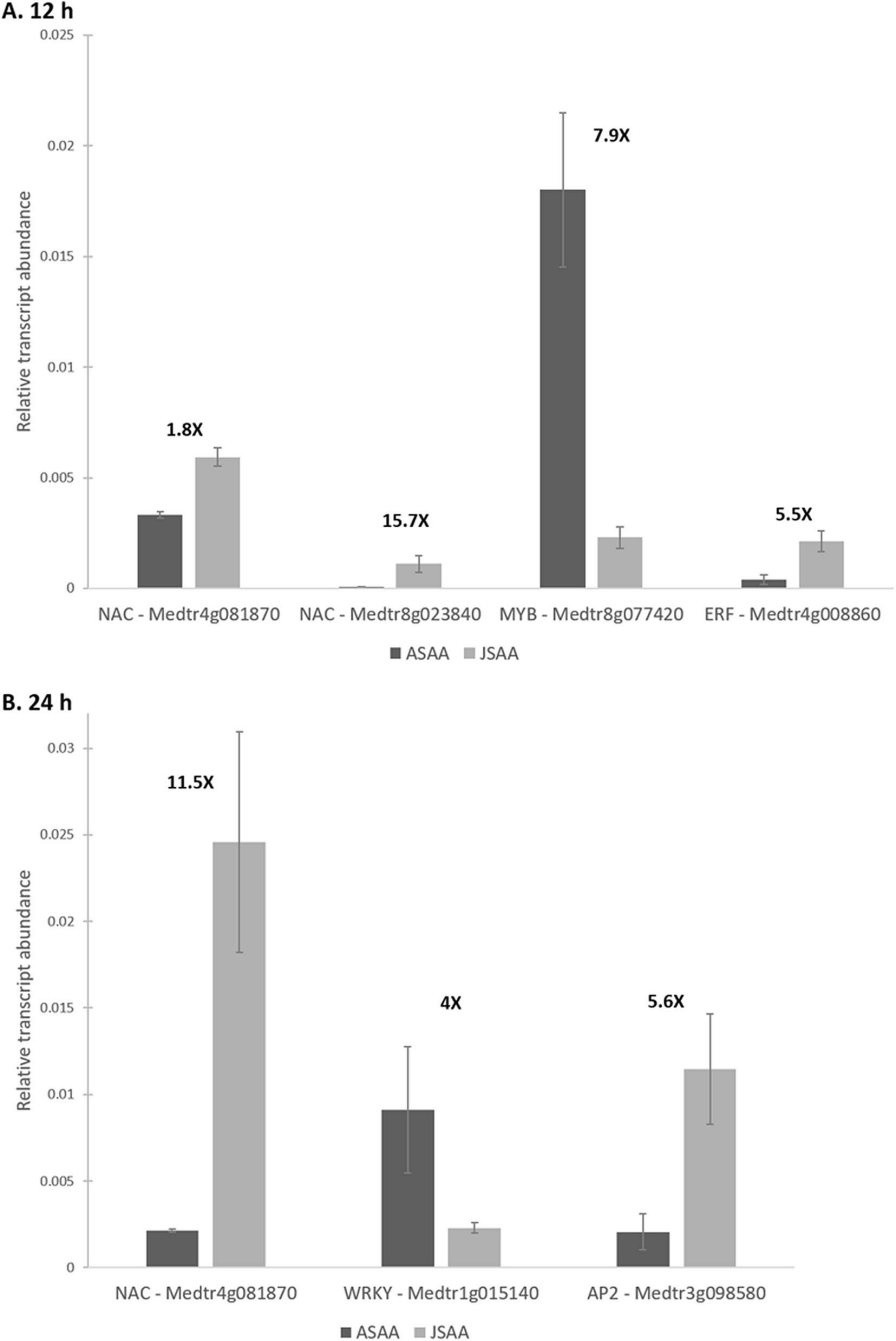
qRT-PCR validation of TF encoding genes. Six TF encoding genes representing different classes were quantified in Jester SAA infested samples (JSAA, light grey) and A17 infested samples (ASAA, dark grey) via qRT-PCR at 12 h (**A**) and 24 h (**B**). The fold changes shown above the bars are in accordance with the fold changes quantified through RNA-sequencing and are significant (*P* < *0.05*, ANOVA). Error bars represent standard errors.

To validate the RNA-seq data, we chose six genes encoding transcription factors that were differentially expressed between Jester and A17 for transcript quantification via qRT-PCR. One NAC TF (*Medtr8g023840*) is up-regulated both at 12 h and 24 h of SAA feeding in the resistant Jester so this gene was tested for both data-sets. A total of three TF (2 NAC TF—*Medtr4g081870, Medtr8g023840* and 1 ERF—*Medtr4g008860*) were significantly (*P* < 0.05) up-regulated in Jester whilst a MYB TF (*Medtr8g077420*) was down-regulated in Jester compared to A17 after 12 h of SAA infestation (Fig. 6A). After 24 h, the NAC (*Medtr4g081870*) and AP2 (*Medtr3g098580*) TFs were up-regulated in Jester whilst the WRKY (*Medtr1g015140*) was up-regulated in A17 (*P* < 0.05) (Fig. 6B). These values are in accordance with the fold-change differences between Jester and A17 quantified via RNA-seq (Supplementary Table 3).

### Generation of a Medicago TILLING population for reverse genetics screening

To determine if any transcription factors identified from our transcriptome profiling were functionally involved in the SAA response, we generated a *Medicago* TILLING population in the cultivar Jester background that could be screened for mutations in candidate genes of interest and phenotyped following SAA infestation. The *M. truncatula* cultivar Jester was selected as the preferred wildtype to generate an EMS derived TILLING population from seed bulked up from a single Jester plant to ensure genetic homogeneity. Jester was selected as it not only harbours resistance to three different aphid species in bluegreen aphid, pea aphid and SAA^29,30^, but also has resistance to *Fusarium oxysporum* f. sp. *medicaginis*^31^, *Phoma medicaginis*^32^ and basal resistance to *Rhizoctonia solani*^33^. Therefore, developing a population in this genetic background could aid the identification of candidate genes through screening mutants in genes in the region of interest fine-mapped for these traits. A suitable EMS dosage rate for our Jester cultivar was first determined by applying EMS at different concentrations ranging from 0% to 0.30%. Triplicate batches of ~ 100 seeds were treated and subsequently the germination percentage of healthy and sick/slow growing plants determined to generate a kill-curve (Supplementary Fig. 2). A noticeable reduction in healthy germinated seedlings was observed at 0.20% EMS dose compared to 0.15% with a drop from 77.6% to 41.2% of healthy seedlings. A similar kill-curve analysis to generate a EMS population in Jester’s near isogenic line A17 by Le Signor^34^ also showed a marked decrease in seedling survival above 0.20%. The fertility of M_1_ plants in the Jester population was 16.8%, which is similar to the 19% observed for 0.15% EMS in the population by LeSignor (2009). Therefore, 0.15% was selected as the concentration to generate the Jester EMS TILLING population, which resulted in a collection of nearly 1,000 M_3_ lines generated via single-seed descent from M_1_ and M_2_s.

### Exome capture sequencing of target genes

To screen the generated Jester TILLING population for mutations in genes of interest, we performed targeted exome capture sequencing. A total of 5,100 M*. truncatula* genes were targeted in 230 lines, representing nearly one-quarter of the lines generated by TILLING. The exome capture probe set targeted genes in the fine-mapped regions of interest for resistance to SAA, *F. oxysporum* and *R. solani*, as well as genes involved in plant defense signalling and terpenoid biosynthesis and the gene target list is shown in Supplementary Table 4. The exome capture data for these 5,100 exome regions ranges from 0.7 Gb to 1.8 Gb where 1 Gb corresponds to over 60 X coverage for 14.3 Mb captured exome regions. On average, 99% of trimmed reads mapped back to the *M. truncatula* genome and 85% of these reads mapped back to exome captured regions. In total, more than 53,600 SNPs were identified in a variety of regions, including exons, introns, splice sites, 5′ and 3′ untranslated regions and resulted in diverse types of outcomes such as missense mutation, loss of start-codon or stop-codon, stop-codon gained, as shown in Supplementary Fig. 3. From over 53,000 SNPs only 959 SNPs led to a pre-mature stop and a high potential for a loss of gene function (Supplementary Table 5). Pre-mature stops were identified in 209 lines out of the 230 lines that were screened, with numbers varying from one to 16. Out of 959 pre-mature stop variations, 471 were C/T variations and 488 were G/A variations.

For twelve genes, a mutation leading to a pre-mature stop-codon was of particular interest as these genes were differentially expressed between the resistant Jester and susceptible A17 cultivars during SAA infestation. Manual sequencing data validation in Integrative Genomics Viewer^35^ of the predicted STOP-calling algorithm reduced this number to nine target genes in nine different TILLING lines. An initial batch of seeds for seven lines was planted out. Assessment of seed viability and poor germination rate resulted in a total of five lines to follow up, each carrying a gene with a loss-of-function mutation that was up-regulated in Jester upon SAA feeding compared to A17. Four of these genes encode for transcription factors: two NAC TFs (*Medtr4g081870* and *Medtr5g014300*), an AP2 domain TF (*Medtr3g098580*) and an ethylene responsive TF (*Medtr7g020980*). The remaining gene (Medtr3g019500) encodes an S-locus lectin kinase family protein.

### SAA infestation on TILLING lines of interest

The *Medicago* TILLING population generated in the resistant cultivar Jester was used as a functional genomics tool to assess the importance of differentially regulated genes in a resistant cultivar in response to aphid infestation. Upregulated genes following SAA infestation in Jester could play an essential role in the plant defense response to aphid infestation. Non-functional copies of such genes identified as premature stop-codons in the TILLING population could potentially have an altered defense response following SAA infestation, leading to enhanced susceptibility and delaying aphid mortality, provided there was no redundancy for these gene functions in SAA resistance. As such, we hypothesize that SAA should survive after 48 h of feeding on this Jester line carrying the loss-of-function gene since resistance is lethal to SAA by two days. Here, we set out to test the five target genes that are significant upregulated in Jester compared to A17 upon SAA feeding (Table 3). Analysis of the sequencing reads in Integrative Genome Viewer showed the STOP mutation was homozygous for the ethylene response TF in the M1146 line whilst for the two NAC TFs, the AP2 TF and the lectin kinase in the M1002, M1087, M1080 and M1027 lines respectively, the STOP mutation is in heterozygous state. Therefore, we screened M_4_ individuals of these four TILLING lines by isolating DNA from individual plant leaf tissue and amplifying the target gene via PCR for sequence analysis. For all lines, three individual plants that carried a homozygous STOP mutation in the target gene were grown to M_5_ seeds and progeny of these plants were kept separate. Plants carrying a homozygous Jester wild-type allele were also kept as a ‘wild-type’ control of the target gene in the same mutational mosaic background of the respective TILLING lines.

**Table 3:** Differentially regulated transcription factors with a premature stop-codon in the corresponding *M. truncatula* TILLING lines in the cultivar Jester genetic background.

Medicago ID	TILLING ID	Description	log FC Jester vs A17 (24 h)^a^
Medtr3g098580	M1080	AP2 domain class transcription factor	2.42
Medtr4g081870	M1002	NAC transcription factor-like protein	3.27
Medtr7g020980	M1146	Ethylene response factor	2.36
Medtr5g014300	M1087	NAC transcription factor-like protein	2.45
Medtr3g019500	M1027	S-locus lectin kinase family protein	1.38

^a^The log fold-change (FC) expression difference between Jester and A17 is shown after 24 h of SAA feeding.

**Figure 7:**
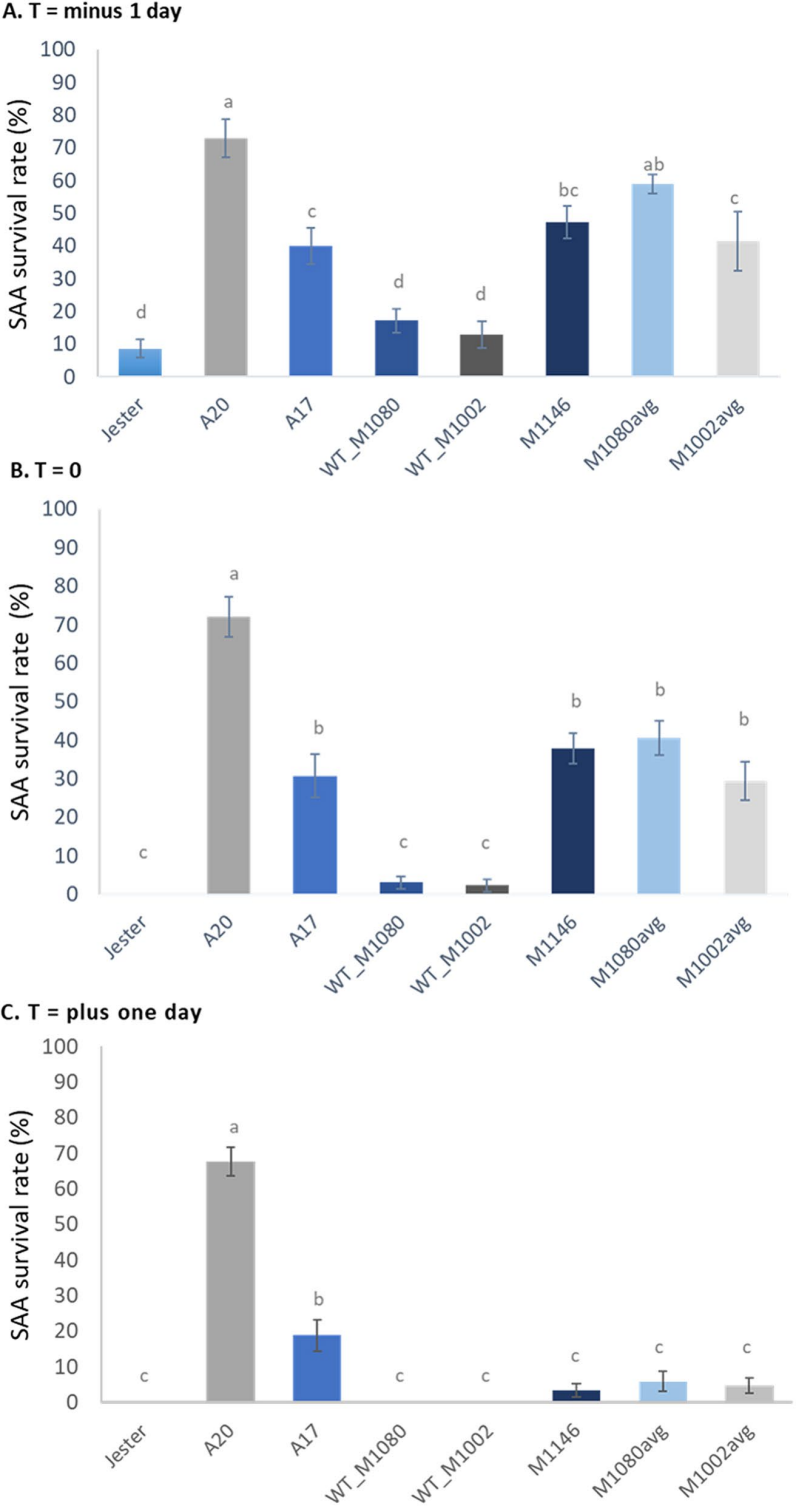
Three truncated TFs cause a delay in SAA mortality compared to their wild-type version in a Jester TILLING background. SAA survival rates were monitored over time and 100% mortality in Jester was set as the null point (T = 0, **B**). The homozygous wild-type allele of our target genes in two TILLING lines (WT_M1080 and WT_M1002) is not significantly different to Jester. However, their three independent homozygous STOP lines that were averaged (M1080avg and M1002avg) as well as the M1146 plants are significantly different from Jester (Tukey–Kramer multiple comparison test, *P* < 0.05) and have increased SAA survival rate at T minus 1 day (**A**) and at T equals 0 (**B**). Although some SAA still survive on the M1146, M1080avg and M1002avg at T plus one day (**C**), this is not significant compared to Jester. Error bars represent standard error and different letters show the significant differences between the samples as determined by Tukey post-hoc testing.

Four-week old plants were infested with SAA and followed for five days. Since biological repeats were performed over time, the SAA colony had slight variations in time for which 100% mortality was reached in all resistant Jester plants. Therefore, we normalized time and state T = 0 when no SAA is alive on Jester, i.e. 100% SAA mortality. Two of the lines, M1087 (NAC TF) and M1027 (lectin kinase family protein) did not show a differential phenotype as they reached 100% SAA mortality concurrently to Jester. Therefore, we focused on the three remaining TILLING lines, M1146, M1080 and M1002, each carrying a truncated TF from a different gene family. For the M1080 and M1002 TILLING lines, progeny (six plants per biological repeat) of three independent homozygous STOP lines were averaged, thereby increasing the EMS mosaic of random mutational background. At T = 0, the TILLING lines with random background mutations but a homozygous wild-type allele for our target gene (WT_M1080 and WT_M1002) were not significantly different to Jester (Fig. 7B). The SAA survival rate on the TILLING lines with a homozygous STOP allele for our gene of interest (either M1146, M1080 or M1002) differed significantly from Jester (*P* < 0.05) for all three genes (Fig. 7A,B). The SAA survival rate on these lines is comparable to A17 at T = 0 suggesting an increased susceptibility of these lines to SAA. However, one day later (T + 1, Fig. 7C), SAA survival rate dropped significantly on the TILLING lines carrying their respective truncated TF and were comparable to Jester (*P* < 0.05) whilst on A17 about 20% of SAA still survived. This delay in mortality rate marks the importance of these TF as regulatory genes involved in SAA resistance.

## Discussion

An in-depth understanding of the molecular mechanisms involved in plant-aphid interactions is needed to combat the rising emergence of aphid insecticide resistance with innovative and viable aphid control strategies. Here, we identified the DEGs in response to SAA feeding in two near-isogenic lines of *M. truncatula*, the resistant (Jester) and susceptible (A17) cultivar. Focusing on two time points, 12 and 24 h after SAA infestation, enabled us to capture the dynamics of these transcriptomic changes during early stages of plant-aphid interactions. To further functionally characterize candidate defense signal transducers and regulators, an EMS TILLING population was generated in the resistant cultivar Jester. This population was screened via targeted exome capture sequencing for premature stop-codons in genes differentially expressed in the resistant and susceptible lines after SAA infestation. This led to the identification of three distinct candidate transcription factors as important molecular players in the defense response against SAA. A premature stop-codon in these genes in a Jester background led to a delay in SAA mortality and enhanced susceptibility compared to their wild-type versions in a similar mosaic of mutational background. Deciphering the molecular changes in this plant-aphid model pathosystem in combination with generating a TILLING population in a resistant cultivar forms a powerful reverse genetics approach to screen and uncover candidate target genes to gain plant resistance against aphid predation.

The molecular changes leading to a resistant or a susceptible defense response following aphid feeding remain poorly understood and in the case of SAA, a major pest of the world number one pasture legume, completely unknown. Here, we made use of the *M. truncatula*-aphid system which has a proven track record as a model to dissect the complex interaction of plant immunity and aphid predation^13^. By comparing and contrasting differentially expressed genes in the resistant Jester and a susceptible A17 genotype, we can distinguish between an overlapping basal defense response and an R-gene induced defense response only initiated in the resistant cultivar Jester through the resistance gene TTR. Although the PCA plot shows distinct clustering of the Jester and A17 (un)infested samples (Fig. 1), a large portion of DEGs upon SAA infestation are shared between Jester and A17 (40%) indicating a highly similar basal resistance (Fig. 2). While the number of regulated genes in A17 upon SAA infestation is comparable to the number of genes regulated in Jester upon SAA feeding and ranges from 14,202 to 15,435 (Table 1), a more than two-fold increase in DEGs is observed over time when comparing Jester to A17. Transcriptomic changes in response to aphid predation that have been characterized thus far in other patho-systems typically identified less DEGs. A study of host and non-host interactions with three aphid species in *Arabidopsis* revealed 874 differentially expressed genes^36^ whilst in barley a total of 974 genes were differentially expressed in comparisons to the no‐aphid control^37^. In maize, infestation with *Rhapalosiphum padi* aphid resulted in over 3000 differentially expressed maize genes after 24 h of aphid predation^38^. Direct comparisons of the number of DEGs between these studies are difficult as they are different plant-aphid interactions and different techniques (microarrays vs RNAseq) and statistical procedures and cut-offs were selected in these studies. To capture those genes that play a major role in *TTR* mediated SAA resistance, we further focused on the DEGs between Jester and A17 after 12 h (2400 genes) and 24 h (5400 genes) of feeding. Interestingly, genes involved in DNA replication are enriched at 12 h whilst this shifts to an enrichment in genes involved in post-translational protein modification and more specifically serine, threonine and tyrosine kinase activity (Fig. 3). This is in line with findings that posttranslational modifications such as protein phosphorylation are an effective means to steer plant immune signaling and induce rapid alterations of pathways to regulate a fast defense response^39^. The mitogen activated protein kinase cascades are a classic example of phosphorylation and activation of subsequent transcription factors allowing transcriptional reprogramming for defense^40^.

The phytohormone involvement of SA and JA in plant defense signaling and specifically against aphids is well documented although their relative contribution and cross‐modulation are still not fully understood^41^. The complexity of hormonal crosstalk increases with diverse plant species regulating different phytohormones in response to distinct aphid species. The octadecanoid pathway was induced exclusively in the resistant Jester *M. truncatula* genotype following bluegreen aphid infestation^23^ whilst cotton aphids on zucchini plants show a transcriptional up-regulation of SA biosynthesis genes^42^. A recent study on legume defense signaling pathways with multiple pea aphid clones shows that the aphid itself manipulates the plant-defense signaling pathways to their own advantage and are able to modulate the SA- and JA- defense signaling pathways^43^. This raises the question whether the prevailing role of SA at the onset of aphid infestation is in effect induced by aphids to antagonistically regulate the JA- signaling pathway and the downstream defense barriers^41,44^ or if it is the plant itself regulating its defense response via the SA pathway^45^. Studham & Macintosh suggested that an activation of another phytohormone, i.e. abscissic acid (ABA), might be triggered by soybean aphid as a decoy strategy to suppress the SA and JA-related defenses in soybean^46^. Our data suggest a highly fine-tuned and complex interaction network not only between SA and JA but also ethylene (ET), ABA, brassinosteroids (BRs) and auxin are involved in the defense response to SAA (Fig. 4). In effect, SA and JA-responsive genes are regulated in both A17 and Jester during aphid infestation and only a handful of genes are differentially regulated when comparing the resistant Jester and susceptible A17 genotype at a given time point during SAA feeding. Whilst auxin related/responsive genes are also regulated in both A17 and Jester, at 24 h after feeding more than 50 auxin-related genes are differentially regulated between Jester and A17 suggesting that recruitment and fine-tuned regulation of auxin signaling is important for resistance to SAA in Jester. Although auxin crosstalk to plant immune networks has been observed^47,48^, there is only one study that previously reported auxin signaling during aphid resistance in the melon—*Aphis gossypii* interaction, as they identified six miRNAs that could potentially regulate auxin interactions^49^. However, rather than a repression of auxin signaling networks, our work provides potential evidence for the recruitment of auxin signaling in response to aphid feeding. Add ABA, BRs and ET phytohormones into the mix and hormonal regulation of plant immunity is a multifaceted complex hub, with ET and auxin acting synergistic to JA and antagonistic to SA^50,51^. The exact role and interaction of auxin in the plant defense network during aphid feeding warrants further investigation and would be a new field to explore to engineer crops with higher resistance against insect pests.

Another promising field to directly impact crop protection is through the study and modulation of transcription factors. After 12 h of SAA feeding, 115 transcription factors are differentially regulated between Jester and A17 whilst this increases to 233 TF encoding genes after 24 h. This corresponds to 4.5% for both time points of the total DEGs between Jester and A17. Over time there is a shift in transcription factor class representation with the percentage of WRKY TFs more than doubling making it the second largest class of TF behind the MYB encoding genes at 24 h (Fig. 5). These are two TF classes for which over-expression of candidate genes has shown enhanced aphid resistance; over-expression of the *CmMYB19* TF in chrysanthemum is shown to improve tolerance against the chrysanthemum aphid (*Macrosiphoniella sanborni*)^52^ and over-expression of *CmWRKY48* was able to inhibit population growth of these aphids^53^. WRKY TFs were also regulated in resistant Jester upon bluegreen aphid infestation with at least one WRKY TF being JA-responsive^54^. Also in tomato, two WRKY TF (WRKY70 and WRKY72) were shown crucial for *Mi-1* R gene mediated resistance against potato aphid^55,56^. Here, the WRKY70 is suppressed by methyl jasmonate whilst TF transcript levels are up-regulated in response to salicylic acid. On the other hand, transcription factors that are down-regulated in Jester upon SAA feeding might be increasing plant susceptibility to aphids when over-expressed. This is the case for the *Arabidopsis* transcription factor MYB102 which activates the ethylene biosynthesis thereby compromising plant defense response against green peach aphid^57^.

We investigated a selection of these TFs from different families further by performing a qRT-PCR validation of six transcription factors over two time points and compared infested Jester to A17 and demonstrate that their regulation is consistent with the RNA-seq data (Fig. 6). To functionally validate some interesting candidate TF genes, we further created a TILLING (Targeting Induced Local Lesions IN Genomes) population of the resistant Jester genotype using 0.15% EMS (Supplementary Fig. 2). TILLING has proven to be useful in functional genomics and combined with advances in next generation sequencing shown to be a valuable reverse genetics strategy^58^. In this study, we screened 230 M_2_ lines using exome capture and next-generation sequencing to catalogue the EMS-induced mutations in 5100 M*. truncatula* genes (Supplementary Table 4, Supplementary Fig. 3)^59^. This highlights the value of a TILLING population since one population can be screened for many targeted genes of interest, i.e. we included genes of interest for *F. oxysporum* and *R. solani*, as well as genes involved in plant defense signalling and terpenoid biosynthesis.

Five TILLING lines contained a potential knockout mutation in a gene that our transcriptome study had shown were differentially up-regulated in Jester compared to A17 following SAA feeding. In each of these genes, the mutation of interest resulted in a premature stop-codon. Four of these genes were transcription factor encoding genes, namely an AP2 domain TF (*Medtr3g098580*) in TILLING line M1080, two NAC TFs (*Medtr4g081870, Medtr5g014300*) in TILLING line M1002 and M1087 respectively and an ethylene response transcription factor (*Medtr7g020980*) in TILLING line M1146 (Table 3). The M1027 TILLING line carried a STOP mutation in *Medtr3g019500*, which encodes for an S-locus lectin kinase family protein. After screening and ensuring homozygosity of the mutation, aphid infestation experiments were set up. Whilst TILLING lines M1027 and M1087 did not show a distinct phenotype compared to Jester, the M1002, M1080 and M1146 lines all showed a significant delay in SAA mortality and enhanced susceptibility to SAA as compared to Jester and to their WT gene versions in a similar mosaic of mutations (Fig. 7). This suggests these three specific transcription factors are important regulatory genes in the defense response to SAA. Twenty-four hours before Jester reached a 100% mortality, plants from lines M1146 and M1002 have a similar aphid survival rate to the moderately susceptible A17 whilst M1080 plants have an aphid survival rate that does not differ significant from the highly susceptible A20 SAA survival rates (Fig. 7A). This raises the question whether the AP2 domain TF is higher up the regulatory network chain than the NAC and ERF TFs. However, at 100% mortality on Jester (T = 0) all three TILLING lines have SAA survival rates in line with the survival rates on A17 plants (Fig. 7B). Only 24 h later do the SAA mortality rates not differ significantly from Jester albeit still not 100% SAA mortality for all three TILLING lines as seen with Jester (Fig. 7C). This delay in mortality potentially marks the importance of these three transcription factors in allowing Jester to acquire a resistant phenotype and hence their significance as regulators in the defence response against SAA. It’s also important to note that a premature STOP mutation of not every TF results in a SAA related mutant phenotype as the disruption of the *Medtr5g014300* NAC TF did not result in delayed SAA mortality. Similarly, an attenuation of R-gene mediated resistance against potato aphid was observed when supressing the tomato *SlWRKY70* gene^55^. The authors concluded that *SlWRKY70* is therefore required for R-gene (*Mi-1*) mediated plant defence response against potato aphid. Given the redundancy known to exist in TF families, it is possible that Medtr5g014300 is still important for SAA resistance but other family members mask its loss. While redundancy may also have masked the full extent of the role of the other three TFs with premature stop codons, it was still possible to observe a clear delay in SAA mortality and enhanced susceptibility, on each of these mutant lines, demonstrating their clear involvement in helping to mediate SAA resistance in the resistant cultivar, Jester. Further research on how these TF contribute to R-gene mediated resistance and which pathways they regulate would be necessary to get an in-depth picture of these TF signalling pathways. In chrysanthemum, researchers recently identified a WRKY TF (*CmWRKY53*) which negatively regulate the resistance to *Macrosiphoniella sanborni* aphids, possibly due to its role in secondary metabolite regulation^60^. Similarly, a MYB transcription factor in Arabidopsis (*MYB102*) was shown to increase plant susceptibility to aphids through the activation of ethylene biosynthesis^57^.

The R-gene providing SAA resistance has been characterized by Klingler et al. (2007) and this *TTR* (*Therioaphis trifolii* resistance) gene was shown to act independently of the *AKR* (*Acyrthosiphon kondoi* resistance) gene to bluegreen aphid, although both genes mapped to a region on chromosome 3. Both A20 and A17 lack the *TTR* gene, however A17 does possess *AIN* (*Acyrthosiphon* induced necrosis) that provides some level of resistance to both bluegreen and pea aphid, but not SAA. The difference in SAA susceptibility between A20 and A17 can be explained by three different quantitative trait loci (QTL) on chromosome three, six and seven^16^. Interaction between the *TTR* gene and other QTL contributing to a difference in aphid susceptibility would be of interest for future research. A recent study showed that two *M. truncatula R* genes against aphids, *AKR* and *AIN*, interact and that these interactions are additive and epistatic^13^.

A growing awareness of (over) use of insecticides combined with rapidly evolving insecticide resistance in aphids highlights the need for the use of non-chemical strategies to control these insect pests. Understanding the molecular and genetic basis of strong resistance to aphids, such as that mediated by the *TTR* gene in *M. truncatula*, will assist the development of resistant cultivars to control aphid populations. This research is the first comparative transcriptome study between a resistant Jester and susceptible A17 *M. truncatula* cultivar to dissect SAA resistance over time. The generation of a Jester TILLING population combined with targeted exome capture and next-generation sequencing allowed for the screening of candidate target genes and marked the importance of three transcription factors in the SAA resistance response in Jester. We believe this study will aid the discovery and utilization of candidate regulatory genes to enhance resistance against SAA and potentially other aphid pests.

## Material & methods

### Aphid rearing and host plants

*Therioaphis trifolii* (Monell) *f. maculata* (spotted alfalfa) aphids were obtained from an asexual, parthenogenetic colony initiated from a single spotted alfalfa aphid clone collected from *Medicago sativa* growing in a field in South Australia. Aphid numbers were maintained by rearing them on caged four-week-old alfalfa (lucerne; *M. sativa*) in natural light in the greenhouse with temperatures ranging from 15 to 30 °C. Aphids were transferred to feeding cages with a fine paintbrush.

### SAA infestation for RNA sequencing

Two closely related genotypes of *M. truncatula* were the primary focus of this study: Jester and A17. The SAA resistant cultivar, Jester, was obtained by recurrent backcrossing to the susceptible Jemalong (A17) genotype, resulting in near isogenic lines which were acquired from the Genetic Resource Centre, the South Australian Research and Development Institute (SARDI). Seeds were scarified, surface disinfected with bleach, germinated and grown as described by Klingler and colleagues^29^. Two mature, fully expanded trifoliate leaves from the primary stem of individual four-week-old plants were infested and caged with 20 adults as described by Gao and colleagues^23^. For the non-infested control plants, two trifoliate leaves of similar developmental stage were caged without aphids. The caged leaves were excised after 12 and 24 h of aphid infestation for both control and aphid-infested Jester and A17 plants. The aphids and leaflets were removed and RNA was extracted from the petioles. Three biological replicates were set up for each aphid‐infested or non-infested control and time point. Each treatment within a biological replicate consisted of petioles from three plants that were pooled to form one sample, yielding a total of six petioles per replicate (from two trifoliate leaves per plant).

### RNA sequencing data analysis

High quality total RNA was isolated using the TRIzol method as described previously^61^ and submitted to the Beijing Genome Institute (BGI) to generate 100 bp Paired-End reads on the V4 Hiseq 2000 platform. Quality of RNA-seq data was checked using FastQC^62^ followed by trimming using Cutadapt^63^ (parameters: –format fastq –overlap 10 –times 3 –minimum-length 25).

Salmon 0.12.0 was used to quantify expression at the transcript level (–validateMappings -l A). Differential gene expression analysis downstream of the RNA-seq quantification was performed with tximport^64^ to import the Salmon files into DEseq2^65^. A DEseq2 standard differential expression analysis was performed using the function *DESeq* and by calling a gene differentially expressed if the adjusted p-value is less than 0.1. To generate the PCA plot, DESeq2 function ‘plotPCA’ was used on the *rlog* transformed counts.

### qRT-PCR

Petiole samples were ground in liquid nitrogen to a fine powder and 150 mg of each ground tissue sample was transferred to 1.5 mL Eppendorf tubes for RNA isolation. Sample was extracted in 500 µL of TRIzol reagent (Invitrogen, Carlsbad, CA) twice with a 15 min incubation at room temperature to homogenize the sample. After 10 min centrifugation (4 °C) at 12 000 g, the supernatant was mixed with 200 µl chloroform, followed by another centrifugation step at 12,000 g and 4 °C for 15 min. Next, the upper phase is transferred to a new Eppendorf tube and is mixed with 300 µL of a high salt precipitation buffer (0.8 M sodium citrate, 1.2 M NaCl) and 300 µL of isopropanol and incubated on ice for at least 10 min to selectively precipitate total RNA. Samples were subsequently centrifuged for 10 min at 12,000 g and 4 °C and the pellet was rinsed twice in 75% ethanol. RNA was dissolved in 50 µl diethylpyrocarbonate (DEPC)-treated water. A total of 1 µg of total RNA was used for first-strand cDNA synthesized as described by^54^. qRT-PCR was performed using a 96-well iCycler (BioRad, Hercules, CA) using thermocycling conditions described previously^23^. Primers were designed using the Primer 3 software^66^ to pick optimal parameters for RT-qPCR conditions (bulletin 2593; Bio-Rad, Hercules, CA). BLASTN analysis of the primer pairs was performed to ensure amplicon specificity. Sequences of each primer pair are listed in Supplementary Table 6. Housekeeping genes to determine relative expression levels were *Actin2* (F: 5′-TCAATGTGCCTGCCATGTATGT-3′, R: 5′-ACTCACACCGTCACCAGAATCC-3′), *Ubiquitin* (F: 5′-GCAGATAGACACGCTGGGA-3′, R: 5′-AACTCTTGGGCAGGCAATAA-3′) and *β-tubulin* (F: 5′-TTTGCTCCTCTTACATCCCGTG-3′, R: 5′-GCAGCACACATCATGTTTTTGG-3′). Threshold cycle (CT) values for all selected genes were normalized to the CT value of three housekeeping genes, whose expression remained constant among various aphid-infested and non-infested tissues. Two technical repeats and three biological repeats were used for data analyses. Relative gene expression was derived from using 2^–ΔCT^, where ΔCT represents the CT of the gene of interest minus the CT of a single housekeeping gene. These values were then averaged across all three housekeeping genes to yield one relative gene expression value. The significance in difference between ratios was analyzed using two-way analysis of variance (ANOVA) at a 5% significance level (*P* < 0.05) using the GraphPad Prism Statistical Software program.

### Medicago TILLING population generation

Four rounds of 0.15% EMS treatment were conducted to generate the EMS Jester population. Seeds were scarified, surface disinfected with bleach and rinsed as described above. EMS treatments per round were conducted on ~ 1,000 seeds placed in a 1 L Schott bottle with 400 mL 0.15% EMS and gently shaken on a rotary shaker. After 24 h the EMS was removed, and seeds rinsed 12 times with 250 mL of H_2_O which incorporated 3 × 30 min soaks. A control treatment (H_2_O only) on 50 seeds was conducted during each EMS round to validate germination rates. Seeds were plated onto moist filter paper (~ 100–200 seeds per Petri dish), sealed with Parafilm, and vernalized at 4 °C in the dark for six days. Plates were subsequently placed at room temperature (~ 21 °C) in the dark for 1–2 days and germination recorded for a subset of plates. An average 77.4% (range from 69–87%) germination rate of healthy M_1_ seedlings was recorded over the four rounds of EMS treatment which was comparable to observations for 0.15% EMS treatment from our previous kill-curve analysis (Supplemental Fig. 1). Healthy seedlings were planted into individual pots and grown in a glasshouse at an average daytime temperature of 29 °C and average night-time temperature of 15 °C. The M_2_ population was generated by single seed descent from M_1_s. To produce the M_2_ population, 15–20 M_2_ seeds were germinated on moist filter paper. Ten seedlings were sown per pot and thinned out after three-four weeks so that only one healthy plant remained in each pot. Leaf material from young leaves of 4–8-week-old plants was retained as the source of material for M_2_ DNA and M_3_ seeds at maturity. M_2_ leaf tissue was harvested in duplicate into deep-well 96-well plates. DNA was extracted from one replicate set using the CTAB method^67^ performed by the Australian Genome Research Facility (AGRF) (Melbourne, VIC). The second set was stored at − 80 °C. On average 4–5 µg of total DNA was obtained for each M_2_ sample at a concentration of 40–50 µg/mL. Upon flowering, foliar tissues from individual M_2_ plants were bagged into onion bags to harvest pods and to prevent cross-pollination. DNA from 230 M_2_ lines and the Jester parental line were used for exome capture.

### Exome capture data analysis

The probe set was designed in consultation with Roche/NimbleGen bioinformatics team, using a relaxed probe set design based on the latest *M. truncatula* genome assembly at the time (Mt4.0; https://phytozome.jgi.doe.gov/pz/portal.html#!info?alias=Org_Mtruncatula). The exome capture sequencing libraries were constructed and sequenced by Novogene (Beijing), using the Roche/NibleGen Exome Capture SeqCap chemistry and the manufacturer’s recommendations. In short, the genomic DNA of each sample was randomly sheared into short fragments of about 350 bp. The obtained fragments were subjected to library construction using the Illumina TruSeq Library Construction Kit, with strictly following the instructions. Briefly, as followed by end repairing, dA-tailing and further ligation with Illumina adapters, the required fragments (300–500 bp in size) with both P5 (5′ AAT GAT ACG GCG ACC ACC GA 3′) and P7 (5′ CAA GCA GAA GAC GGC ATA CGA GAT 3′) primer sequences were PCR amplified. After gel electrophoresis and subsequent purification, the required fragments were obtained for library construction. Paired-End sequencing was performed on Illumina HiSeq platform, with the read length of 150 bp at each end. A full list of target genes can be found in Supplementary Table 4.

### Paired end read-mapping

Paired-end reads were trimmed for Illumina adapter sequences with reads trimmed to less than 25 bp discarded using Cutadapt^63^ (parameters: –format fastq –overlap 10 –times 3 –minimum-length 25). Trimmed reads were then mapped to *Medicago truncatula* genome assembly (version 4.0)^68^ using BWA (v0.7.15)^69^. BAM files generated from BWA output were sorted and indexed using SAMtools version 0.1.19^69^.

### Variant calling and annotation

SAMtools mpileup was used to call variants on BAM files of each line. VCF files generated in previous step were filtered for EMS mutations using UNIX awk utility. EMS variants were annotated using SnpEff^70^.

### Screening TILLING lines of interest

Manual validation of premature stop-codons in Integrative Genomics Viewer^71^ resulted in a final selection of seven TILLING lines (M_3_ seed) that were initially planted out. 10 pods per TILLING line were broken up and individual seed was scarified with sandpaper. Seeds were plated on moist filter paper (~ 30 seeds per petri dish) sealed and vernalized at 4 °C in the dark for 3 days. After the plates were placed at ambient temperature for 2 days, the seedlings were transferred to individual pots and grown in a controlled growth room with 25 °C average day and 15 °C average night temperature. A combination of factors such as few seed, poor germination/growth/survival rate resulted in a total of five lines (M1002, M1080 and M1146, M1087, M1027) that we followed up with PCR screening and sequencing of the target genes. Therefore, a second batch was planted out and a trifoliate leaf of thirty plants per M_4_ TILLING line was harvested for DNA extractions. DNA from individual plant leaf tissue was extracted using the DNeasy Plant Mini Kit (Qiagen, Hilden, Germany) according to manufacturer’s instructions. Subsequent PCR amplification of the target genes in the corresponding TILLING lines was performed at 98 °C for 3 min, followed by 35 cycles of 98 °C for 15 s, 63 °C for 25 s, and 72 °C for 50 s, finishing with an extension step at 72 °C for 10 min. Primers were designed surrounding the SNP position of interest and the amplified fragment ranged from 490 to 622 bp. A list of the corresponding primers for *Medtr4g081870* in TILLING line M1002, *Medtr3g098580* in TILLING line M1080 and *Medtr7g020980* in TILLING line M1146, *Medtr5g014300* in line M1087, *Medtr3g019500* in line M1027 can be found in Supplementary Table 7. PCR products were run on a 1% agarose gel in TAE buffer for 40 min at 90 V to verify purity and size. Isolation of the PCR products was performed via excision of the single band of the expected size for each gene target and purified with the Qiaquick gel extraction kit (Qiagen). All samples with a band were sent off to the Australian Genome Research Facility (AGRF, Melbourne, VIC) for Sanger sequencing. Results were analysed and plants with homozygous mutation leading to a premature stop-codon were retained for testing with SAA, as well as plants with a homozygous wild-type allele i.e. with no mutation.

### SAA infestation on TILLING lines of interest

Six SAA were placed on a single trifoliate leaf and caged as described before^9^ and mortality was recorded for five days. For the M1087, M1027, M1080 and M1002 TILLING lines, progeny of a total of three independent lines with a homozygous STOP mutation in the respective target gene were tested and averaged, thereby increasing the mutational background. For each of these homozygous STOP lines, a total of six plants were tested, bringing the total to eighteen plants per TILLNG line per biological repeat. Progeny of plants carrying a homozygous wild-type allele for the target genes in M1080 and M1002 were pooled and a total of 15 plants were infested per biological repeat. This is the same number of plants for the controls (Jester, A17, A20) and the M1146 TILLING line. In summary, the survival rates of a total of 90 aphids over fifteen plants was followed and averaged for the M1146, wild-type M1080 and wild-type M1002 lines as well as the Jester, A17 and A20 controls, whilst this was 108 aphids over eighteen plants for the homozygous STOP M1080, M1087, M1027 and M1002 lines. Survival rate was recorded and to normalize for the slight variations of the SAA colony over time for the three biological repeats, we set time point equals zero when 100% mortality rate was reached in all resistant plants. Statistical differences between the lines were calculated by ANOVA with post-hoc Tukey HSD testing (*P* < 0.05).

## Supplementary Information

Supplementary Data.

Supplementary Table 1.

Supplementary Table 2.

Supplementary Table 4.

Supplementary Table 5.
