# Supplementary material for: A functional genomics approach to dissect spotted alfalfa aphid resistance in Medicago truncatula
Source: Sci Rep. 2020 Dec 17;10:22159. doi: 10.1038/s41598-020-78904-z (PMC7746763; doi:10.1038/s41598-020-78904-z)

A functional genomics approach to dissect spotted alfalfa aphid resistance in *Medicago truncatula*

Silke Jacques[1,2], Jana Sperschneider[3], Gagan Garg[1], Louise F. Thatcher[4], Ling-Ling Gao[1], Lars G. Kamphuis[1,2,5], Karam B. Singh[1,2,5*]

[1] CSIRO Agriculture and Food, Floreat, WA 6014, Australia

[2] Curtin University, Centre for Crop and Disease Management, Bentley, WA 6102, Australia

[3] Biological Data Science Institute, The Australian National University, Canberra, ACT 2600, Australia

[4] CSIRO Agriculture and Food, Canberra, ACT 2601, Australia

[5] The UWA Institute of Agriculture, University of Western Australia, Crawley, WA 6009, Australia

[*] Author for correspondence: karam.singh@csiro.au; phone: +61 8 9333 6320; fax: +61 8 9387 8991

Supplementary Information File

This file contains 3 supplementary figures and 7 supplementary tables.

List of Legends

Supplementary Figure 1. Quantification of defence marker gene PR10 and resistant specific lipoxygenase (LOX) gene.

Quantitative polymerase chain reaction (qPCR) shows the spotted alfalfa (SAA) aphid infestation was successful as the pathogenesis related 10 (PR10) gene was upregulated in both susceptible and resistant cultivar compared to non-infested controls. The Jester-specific induction of the lipoxygenase gene (LOX) is apparent after 24 h of infestation.

Supplementary Figure 2. Deciding optimal EMS concentration through mutagenesis killing curve

Different concentrations of EMS ranging from 0 to 0.30 % were applied to Jester seeds and the germination rate was recorded as well as the percentage of healthy looking and sick/slow growing plants. The optimal EMS dose to generate a Jester TILLING population was chosen as 0.15 % since an increase to 0.20 % EMS results in only 41 % of healthy plants. Similar results were obtained in two other independent dosage experiments.

Supplementary Figure 3. Mutation counts and percentages by type and by region in the *Medicago truncatula* cultivar Jester TILLING population.

The counts and percentages are shown per mutation type, or as the region in the gene where the mutation occurred. The lower panel displays the mutation percentages per genetic region as a bar chart.

Supplementary Table 1. Differentially expressed *Medicago truncatula* genes after 12 h of SAA feeding

baseMean = the average of the normalized counts taken over all samples; log2FoldChange = log2 fold change between the groups; lfcSE = standard error of the log2FoldChange estimate; stat = Wald statistic; pvalue = Wald test p-value; padj = Benjamini-Hochberg adjusted p-value

Supplementary Table 2. Differentially expressed *Medicago truncatula* genes after 24 h of SAA feeding *baseMean = the average of the normalized counts taken over all samples; log2FoldChange = log2 fold change between the groups; lfcSE = standard error of the log2FoldChange estimate; stat = Wald statistic; pvalue = Wald test p-value; padj = Benjamini-Hochberg adjusted p-value*

Supplementary Table 3. Differential expression values of selected transcription factors in the RNAseq data set.

a. The log 2 fold changes (FC) of Jester infested with SAA (JSAA) and A17 infested with SAA (ASAA) are shown

Supplementary Table 4. Probe set list of *Medicago truncatula* genes for exome capture sequencing.

The total 6380 genes used for exome capture sequencing are shown with their Medicago identifiers (ID) and their respective gene function and their chromosomal location.

Supplementary Table 5. TILLING lines carrying a gained STOP mutation.

The SNP position on chromosome is shown with the reference (REF) basepair altered (ALT) to the mutated basepair due to EMS treatment, resulting in a STOP codon.

Supplementary Table 6. Forward and reverse primer pairs used for validation RT-qPCR of regulated *Medicago truncatula* transcription factors.

Forward and reverse primer sequences used for RT-qPCR validation of transcription factors.

Supplementary Table 7. Primers used to multiply the respective genes of interest via PCR for subsequent sequencing in the corresponding *Medicago truncatula* TILLING lines.

Forward and reverse primer sequences used for PCR validation of the TILLING lines of interest.

Supplementary Tables

Supplementary Table 3. Differential expression values of selected transcription factors in the RNAseq data set.

Medicago gene ID	Description	Time point	log2 FC JSAA – ASAA[a]	Adjusted p-value
Medtr4g081870	NAC transcription factor-like protein	12 h	2.08	4.31E-07
Medtr8g023840	NAC transcription factor-like protein	12 h	1.83	3.19E-04
Medtr8g077420	MYB family transcription factor	12 h	-3.80	1.86E-15
Medtr4g008860	ethylene response factor	12 h	2.11	1.19E-06
Medtr4g081870	NAC transcription factor-like protein	24 h	3.27	2.30E-19
Medtr1g015140	WRKY family transcription factor	24 h	-1.60	2.72E-06
Medtr3g098580	AP2 domain class transcription factor	24 h	2.42	5.05E-04

a. The log 2 fold changes (FC) of Jester infested with SAA (JSAA) and A17 infested with SAA (ASAA) are shown

Supplementary Table 6. Forward and reverse primer pairs used for validation RT-qPCR of regulated *Medicago truncatula* transcription factors.

Medicago gene ID	Description	Forward primer sequence	Reverse primer sequence
Medtr4g081870	NAC TF	GGGTGAGGCCTAATAGAGCA	AAGGGCTTTACCCTCATGGA
Medtr8g023840	NAC TF	TGTGATGTAGCTGTTTTTCTCTTCC	GGTGGCTGGTCTTGGATGAT
Medtr8g077420	MYB TF	TGGACTCCAAGGCAAAACAAA	GACCATGCTCAATGCGCCTA
Medtr4g08860	Ethylene Responsive TF	TCACTTTCCCTTCACTCTTCAAC	GCTTCTGTTTCACCGCACG
Medtr1g015140	WRKY TF	ACCAGCAGTGGCCATTTGAA	GGCTACCACCTGCTACTTCG
Medtr3g098580	AP2 domain TF	GCTCTTACACAAGTCATGGGC	CTGTGGTGGCTGAGATTGTTC

Supplementary Table 7. Primers used to multiply the respective genes of interest via PCR for subsequent sequencing in the corresponding *Medicago truncatula* TILLING lines.

Medicago gene ID	TILLING ID	Forward primer	Reverse Primer
Medtr3g098580	M1080	ATGACTATGGTGCATATACGTACC	ATATGTTTCTCGAGAAAAGTGTTG
Medtr4g081870	M1002	CAAGGCCATGCAACTACA	CATTTTCCTCAAACTCAGATTT
Medtr7g020980	M1146	TGCATGTGATGTTAATGAAAG	GATTGTACAAACACCGATATCTC
Medtr5g014300	M1087	CTTGCAGTACGGTTCAGGGA	TTGACTTTTACCTCCGAGCC
Medtr3g019500	M1027	ACTCCACACTTACCAACACACA	ACAAATGGACATCAAGTATCCCA

Supplementary Figures

Supplementary Figure 1. Quantification of defence marker gene PR10 and resistant specific lipoxygenase (LOX) gene.

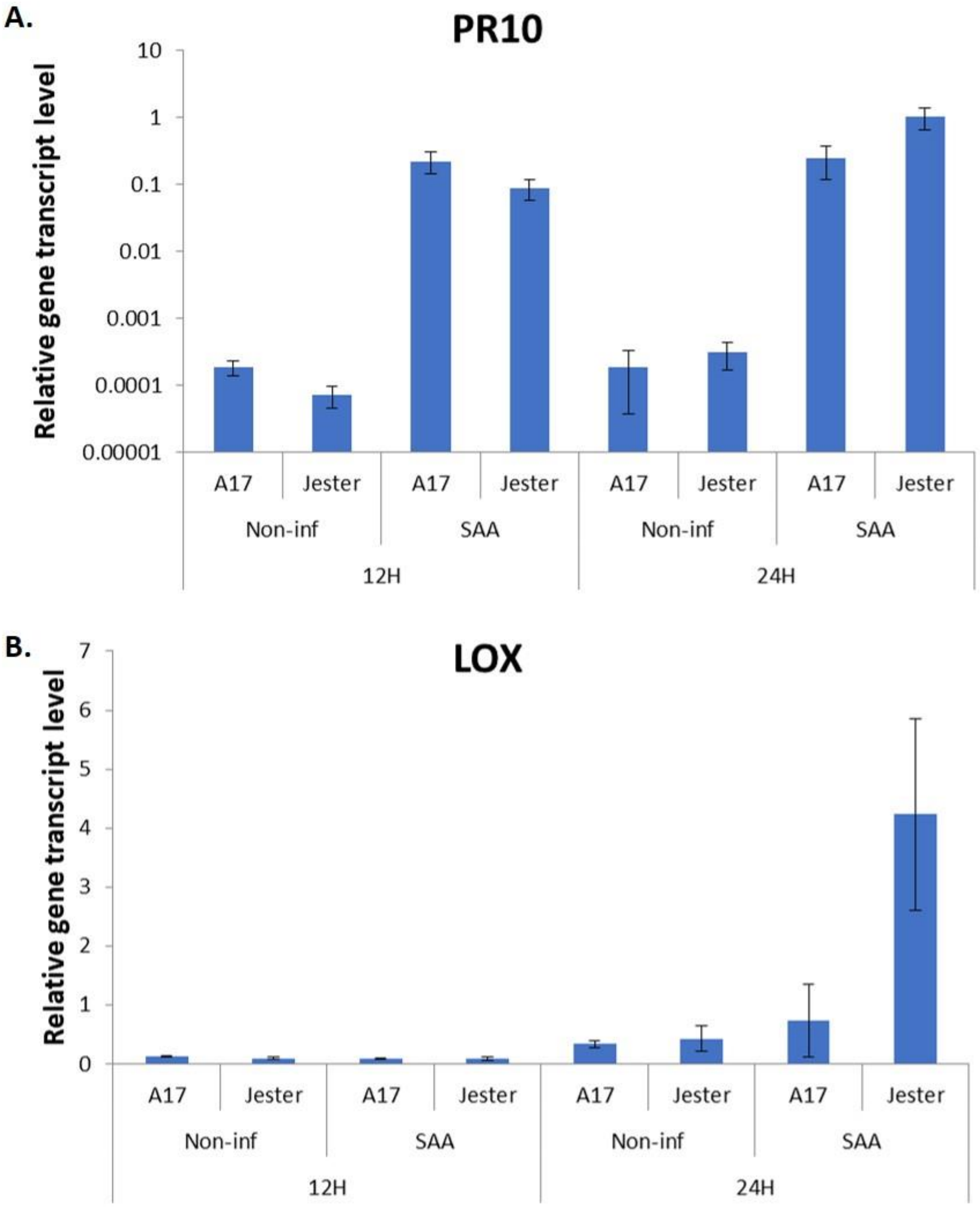

Supplementary Figure 2. Deciding optimal EMS concentration through mutagenesis killing curve

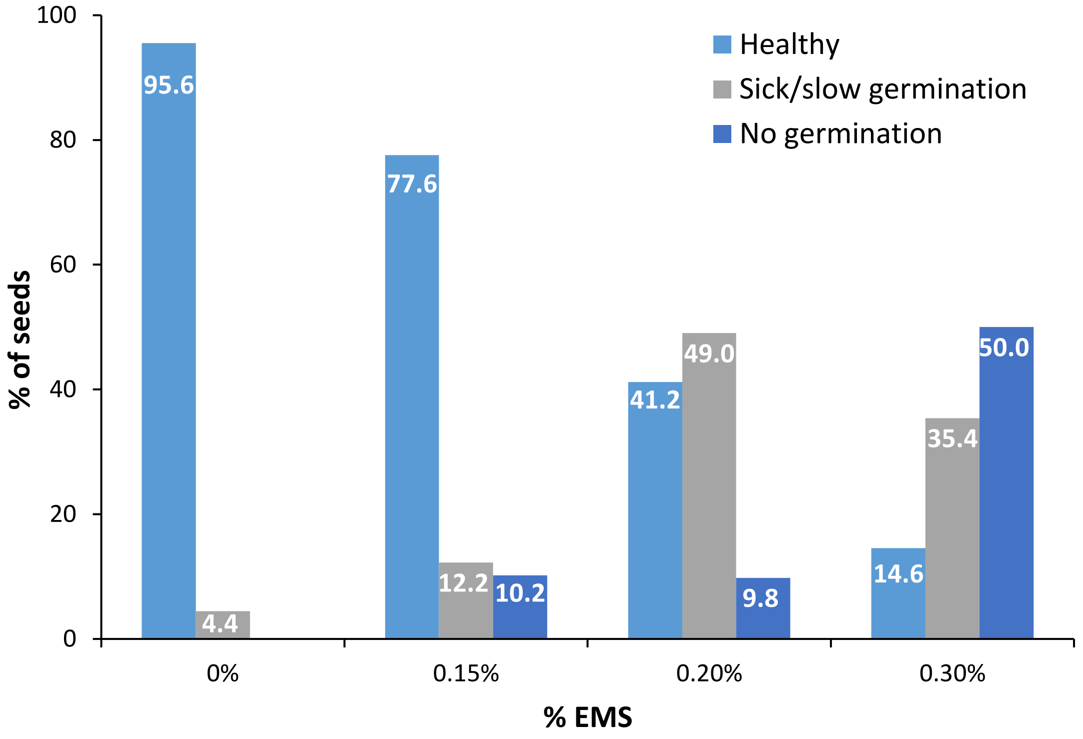

Supplementary Figure 3. Mutation counts and percentages by type and by region in the *Medicago truncatula* cultivar Jester TILLING population.

Number of effects by type and region

Type

Type (alphabetical order)	Count	Percent
3_prime_UTR_variant	4,188	3.233%
5_prime_UTR_premature_start_codon_gain_variant	366	0.283%
5_prime_UTR_variant	3,438	2.654%
downstream_gene_variant	32,988	25.466%
intergenic_region	330	0.255%
intron_variant	33,091	25.546%
missense_variant	18,695	14.432%
non_coding_transcript_exon_variant	37	0.029%
splice_acceptor_variant	153	0.118%
splice_donor_variant	189	0.146%
splice_region_variant	1,187	0.916%
start_lost	71	0.055%
stop_gained	1,158	0.894%
stop_lost	24	0.019%
stop_retained_variant	35	0.027%
synonymous_variant	7,549	5.828%
upstream_gene_variant	26,038	20.101%

Region

Type (alphabetical order)	Count	Percent
DOWNSTREAM	32,988	25.754%
EXON	27,432	21.417%
INTERGENIC	330	0.258%
INTRON	32,120	25.077%
SPLICE_SITE_ACCEPTOR	153	0.119%
SPLICE_SITE_DONOR	189	0.148%
SPLICE_SITE_REGION	846	0.66%
UPSTREAM	26,038	20.328%
UTR_3_PRIME	4,188	3.27%
UTR_5_PRIME	3,804	2.97%

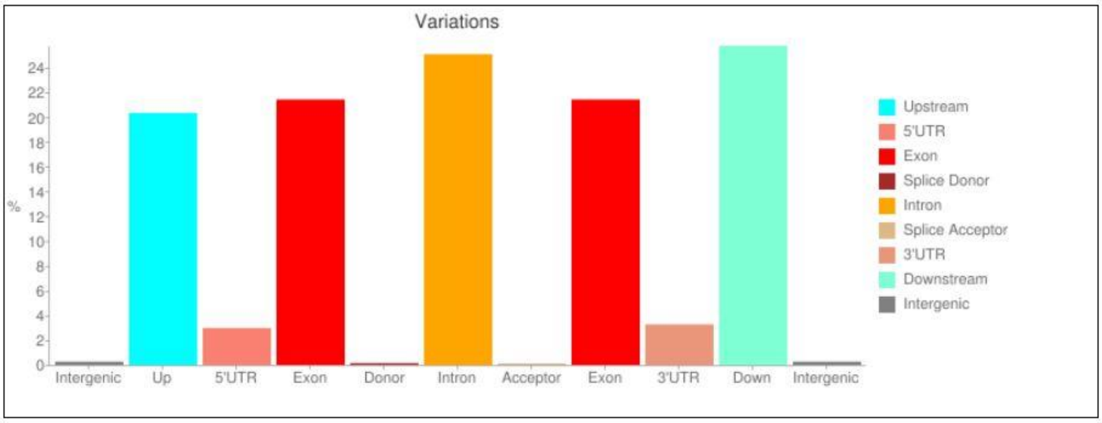

Supplement: Supplementary file 5 — Supplementary Table 5. [file 41598_2020_78904_MOESM5_ESM.pdf]
